# Supplementary material for: Identification of photoperiod-induced specific miRNAs in the adrenal glands of Sunite sheep (Ovis aries)
Source: Front Vet Sci. 2022 Jul 22;9:888207. doi: 10.3389/fvets.2022.888207 (PMC9354845; doi:10.3389/fvets.2022.888207)
Supplement: Supplementary file 1 [file Data_Sheet_1.ZIP › Supplementary file/miRDeep2 file.zip!/miRDeep2 file/Novel_Structure/LP42A2/Novel_249.pdf]

Provisional ID	:	NC_019463.2_13627
Score total	:	2.3
Score for star read(s)	:	-1.3
Score for read counts	:	0
Score for mfe	:	2
Score for randfold	:	1.6
Score for cons. seed	:	
Total read count	:	839
Mature read count	:	839
Loop read count	:	0
Star read count	:	0

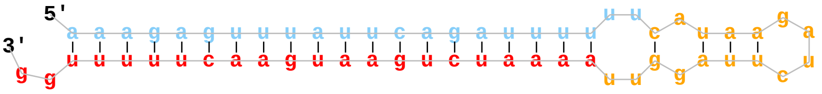

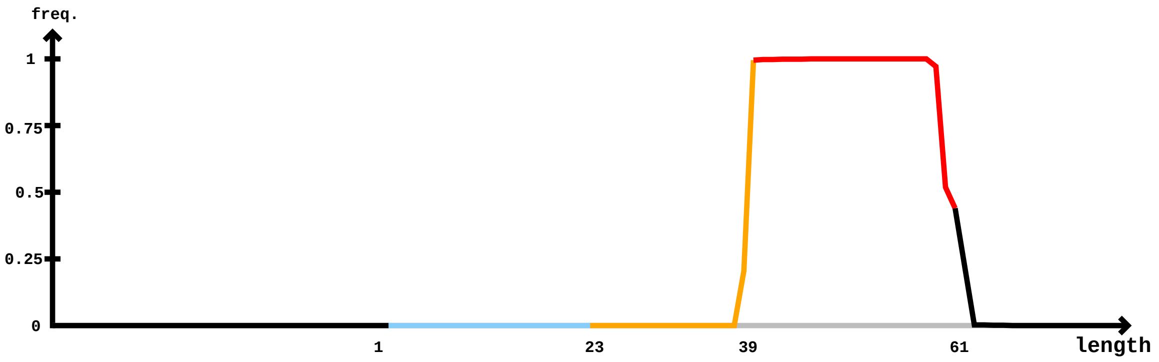

Star Mature

```
5'- uacuauuaacucuaaauaguuaauagguuggccaaagaguuuauucagauuuuuucauaagaucuuagguuaaaaucugaaugaacuuuuuggccaaguuacuaauuuaa  -3'   exp
    ..(((((((((......)))))))))).((((((((((((((((((((((((((..(.(((....))).)..)))))))))))))))))))))))))).............    reads   mm   sample
    ......................................................................uaaaaucugaaugaacuuuu....................    1       0    seq
    ......................................................................Aaaaaucugaaugaacuuuuu...................    111     1    seq
    ......................................................................Gaaaaucugaaugaacuuuuug..................    1       1    seq
    ......................................................................Aaaaaucugaaugaacuuuuug..................    59      1    seq
    .......................................................................aaaaucugaaugaacuuuu....................    23      0    seq
    .......................................................................aaaaucugaaugaacuuuCu...................    2       1    seq
    .......................................................................aaaaucugaaugaacuuuuG...................    2       1    seq
    .......................................................................aaaaucugaaugaacuuuuu...................    258     0    seq
    .......................................................................aaaaucugaaCgaacuuuuu...................    6       1    seq
    .......................................................................aaaaucugaaugaacuuuuGg..................    1       1    seq
    .......................................................................aaaaucugaaugaacuuuuuC..................    3       1    seq
    .......................................................................aaaaucugaaugaGcuuuuug..................    1       1    seq
    .......................................................................aaaaucAgaaugaacuuuuug..................    1       1    seq
    .......................................................................aaaaucugaaugaacuuuuGgg.................    1       1    seq
    .......................................................................aaaaucugaaugaGcuuuuugg.................    1       1    seq
    .......................................................................aaaCucugaaugaacuuuuugg.................    11      1    seq
    .......................................................................aaaaucugaaugaacuuuuugg.................    291     0    seq
    .......................................................................aaaaCcugaaugaacuuuuugg.................    42      1    seq
    .......................................................................aaaaucugaaugaacuuuuuggc................    2       0    seq
    .......................................................................aaaaucugaaugaacuuuuuggU................    18      1    seq
    ........................................................................aaaucugaaugaacuuuuu...................    1       0    seq
    ........................................................................aaaucugaaCgaacuuuuugg.................    1       1    seq
    ..........................................................................auUugaaugaacuuuuuggcca..............    1       1    seq
    .............................................................................ugaGugaacuuuuuggccaag............    1       1    seq
```
